# Benefits of Practical Phrenology

**Published:** 1835

**Authors:** 


### IV.—Benefits of Practical Phrenology.

A couple of "down-easters" are consoling themselves under the death of Dr. Spurzhiem, by making a practical application of the doctrines which he sought to naturalize in their native country. They were lately in our good city, the Gotham of the West, and opened a shop where they wrote out characters at one dollar per head, which was certainly dog cheap. When one failed to give a response which was grateful to the "self-esteem" of the pilgrim to this new Delphic Oracle, the other was always at hand to correct his mistakes, by a second manipulation. Printed blanks were kept and filled up with the numbers indicated on the arc of the callippers, so that each one bore away his character and destiny in his breeches pocket! Thus many of our citizens have undergone a kind of graduation, and are furnished with diplomas as conscientious, pious, benevolent or loving gentlemen, which may be used as future exigencies require. We have not heard that the organ of "credulity" was particularly developed in any of them, but it is well known that the defects of extensity are supplied by the strength of intensity. Time, the great teacher, can only reveal to us the fruits of this scientific mission to the benighted West; but we may anticipate that they will be "pretty considerable:" That many new projects will be set on foot, by those who have been apprized that they are endowed with "ideality"—that the "combative" will indulge their instict under a new warrant—that the "acquisitive" will branch out into additional speculations—and bachelors, reassured, make new overtures, backed by a certificate.

Could those distinguished and dignified philosophers, Gall and Spurzhiem, have foreseen such a prostitution as we have lately witnessed of the principles to which they devoted their lives, all their "firmness" could not have enabled them to die in peace.

